# *N*-Benzyl­pyridin-2-amine

**DOI:** 10.1107/S1600536810044351

**Published:** 2010-11-06

**Authors:** Gai Gai Wang, Hong Zhao

**Affiliations:** a School of Chemistry and Chemical Engineering, Southeast University, Nanjing 210096, People’s Republic of China

## Abstract

In the title compound, C_12_H_12_N_2_, the dihedral angle between the benzene and pyridine rings is 67.63 (8)°. Mol­ecules are linked into centrosymmetric dimers by a simple inter­molecular N—H⋯N hydrogen bond with graph-set motif *R*_2_^2^(8).

## Related literature

For the application of Schiff base compounds in coordination chemistry, see: Garnovskii *et al.* (1993 ▶); Gong & Xu (2008 ▶). For the synthesis, see: Xu *et al.* (2009 ▶). For graph-set notation of hydrogen bonds, see: Bernstein *et al.* (1995 ▶). For another report on the structure of *N*-benzyl­pyridin-2-amine, see: Wang *et al.* (2010 ▶).
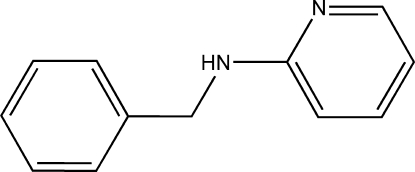


## Experimental

### 

#### Crystal data

C_12_H_12_N_2_
*M* *_r_* = 184.24
Triclinic, 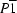

*a* = 5.9233 (10) Å
*b* = 8.0984 (15) Å
*c* = 10.602 (2) Å
α = 94.916 (15)°
β = 91.36 (1)°
γ = 94.451 (15)°
*V* = 504.95 (16) Å^3^
*Z* = 2
Mo *K*α radiation
μ = 0.07 mm^−1^
*T* = 295 K
0.25 × 0.20 × 0.18 mm

#### Data collection

Rigaku SCXmini diffractometer
Absorption correction: multi-scan (*CrystalClear*; Rigaku, 2005 ▶) *T*_min_ = 0.980, *T*_max_ = 0.997
4612 measured reflections
1955 independent reflections
1039 reflections with *I* > 2σ(*I*)
*R*_int_ = 0.062

#### Refinement

*R*[*F* ^2^ > 2σ(*F* ^2^)] = 0.076
*wR*(*F* ^2^) = 0.183
*S* = 1.06
1955 reflections
127 parameters
H-atom parameters constrained
Δρ_max_ = 0.14 e Å^−3^
Δρ_min_ = −0.16 e Å^−3^

### 

Data collection: *CrystalClear* (Rigaku, 2005 ▶); cell refinement: *CrystalClear*; data reduction: *CrystalClear*; program(s) used to solve structure: *SHELXS97* (Sheldrick, 2008 ▶); program(s) used to refine structure: *SHELXL97* (Sheldrick, 2008 ▶); molecular graphics: *SHELXTL/PC* (Sheldrick, 2008 ▶); software used to prepare material for publication: *SHELXTL/PC*.

## Supplementary Material

Crystal structure: contains datablocks I, global. DOI: 10.1107/S1600536810044351/bx2323sup1.cif

Structure factors: contains datablocks I. DOI: 10.1107/S1600536810044351/bx2323Isup2.hkl

Additional supplementary materials:  crystallographic information; 3D view; checkCIF report

## Figures and Tables

**Table 1:** Hydrogen-bond geometry (Å, °)

*D*—H⋯*A*	*D*—H	H⋯*A*	*D*⋯*A*	*D*—H⋯*A*
N1—H1*A*⋯N2^i^	0.86	2.26	3.070 (3)	158

Symmetry code: (i) 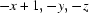.

**Fig. 1.**
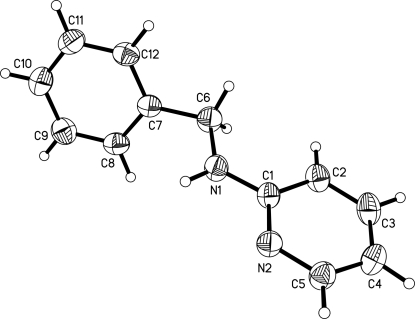
The molecular structure of the title compound, showing the atomic numbering scheme. The displacement ellipsoids are drawn at the 30% probability level.

**Fig. 2.**
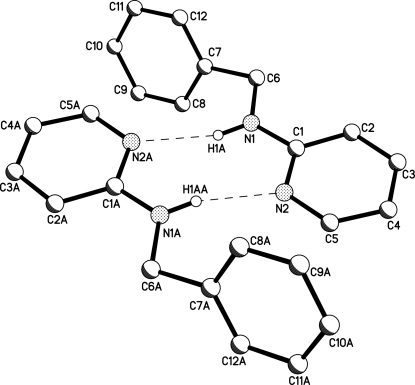
The diagram of the dimer linked by the intermolecular hydrogen bonds. The H atoms not involved in hydrogen bonds have been omitted for charity. Symmetry code: (a) -x + 1, -y, -z.

## supplementary crystallographic information

### Comment

Schiff base compounds have attracted great attention due to their application in coordination chemistry (Garnovskii *et al.*, 1993; Gong & Xu, 2008), and also offer a simple method of synthesis novel amine compounds. The title compound is synthesized from the Schiff base (*E*)-*N*-benzylidenepyridin-2-amine, and the crystal structure is reported here.

In the molecule of the title compound (Fig. 1) bond lengths and angles have normal values. The dihedral angle between the benzene ring and pyridine ring is 67.63 (8)°. In the solid state the molecules are linked into centrosymmetric dimers by a simple N—H···N interaction with set graph-motif R_2_^2^(8) (Bernstein *et al.*, 1995), (Fig. 2; Table 1).

### Experimental

The (*E*)-*N*-benzylidenepyridin-2-amine was prepared from benzaldehyde and pyridin-2-amine according to the reported method (Xu *et al.*, 2009). To a mixture of (*E*)-*N*-benzylidenepyridin-2-amine (20 mmol), NaBH_4_ (100 mmol) in 1,4-dioxane (50 ml), acetic acid (100 mmol) in 1,4-dioxane was added dropwise at 0°C. Then the mixture was heated at 120°C for 2 h then cooled and the solvent removed under vacuum. The residue was poured into water (20 ml) and extracted with chloroform three times (50 ml). The extract was dried (CaCl_2_) and the solvent removed under vacuum to give the crude title compound. Pale yellow crystals suitable for X-ray analysis were obtained by slow evaporation of a 95% ethanol/water solution.

### Refinement

All H atoms were detected in a difference map, but were placed in calculated positions and refined using a riding motion approxmation, with C—H = 0.93–0.97 Å, with *U*_iso_(H) = 1.2*U*_eq_(C); N—H = 0.86 Å, with *U*_iso_(H) = 1.2*U*_eq_(N).

### Crystal data

**Table:**

C_12_H_12_N_2_	*Z* = 2
*M**_r_* = 184.24	*F*(000) = 196
Triclinic, *P*1	*D*_x_ = 1.212 Mg m^−^^3^
Hall symbol: -P 1	Mo *K*α radiation, λ = 0.71073 Å
*a* = 5.9233 (10) Å	Cell parameters from 904 reflections
*b* = 8.0984 (15) Å	θ = 2.5–27.4°
*c* = 10.602 (2) Å	µ = 0.07 mm^−^^1^
α = 94.916 (15)°	*T* = 295 K
β = 91.36 (1)°	Block, pale yellow
γ = 94.451 (15)°	0.25 × 0.20 × 0.18 mm
*V* = 504.95 (16) Å^3^	

### Data collection

**Table:**

Rigaku SCXmini diffractometer	1955 independent reflections
Radiation source: fine-focus sealed tube	1039 reflections with *I* > 2σ(*I*)
graphite	*R*_int_ = 0.062
Detector resolution: 13.6612 pixels mm^-1^	θ_max_ = 26.0°, θ_min_ = 3.1°
CCD profile fitting scans	*h* = −7→7
Absorption correction: multi-scan (*CrystalClear*; Rigaku, 2005)	*k* = −9→9
*T*_min_ = 0.980, *T*_max_ = 0.997	*l* = −13→13
4612 measured reflections	

### Refinement

**Table:**

Refinement on *F*^2^	Primary atom site location: structure-invariant direct methods
Least-squares matrix: full	Secondary atom site location: difference Fourier map
*R*[*F*^2^ > 2σ(*F*^2^)] = 0.076	Hydrogen site location: inferred from neighbouring sites
*wR*(*F*^2^) = 0.183	H-atom parameters constrained
*S* = 1.06	*w* = 1/[σ^2^(*F*_o_^2^) + (0.0676*P*)^2^] where *P* = (*F*_o_^2^ + 2*F*_c_^2^)/3
1955 reflections	(Δ/σ)_max_ < 0.001
127 parameters	Δρ_max_ = 0.14 e Å^−^^3^
0 restraints	Δρ_min_ = −0.15 e Å^−^^3^

### Special details

**Table:**

Geometry. All e.s.d.'s (except the e.s.d. in the dihedral angle between two l.s. planes) are estimated using the full covariance matrix. The cell e.s.d.'s are taken into account individually in the estimation of e.s.d.'s in distances, angles and torsion angles; correlations between e.s.d.'s in cell parameters are only used when they are defined by crystal symmetry. An approximate (isotropic) treatment of cell e.s.d.'s is used for estimating e.s.d.'s involving l.s. planes.
Refinement. Refinement of *F*^2^ against ALL reflections. The weighted *R*-factor *wR* and goodness of fit *S* are based on *F*^2^, conventional *R*-factors *R* are based on *F*, with *F* set to zero for negative *F*^2^. The threshold expression of *F*^2^ > σ(*F*^2^) is used only for calculating *R*-factors(gt) *etc*. and is not relevant to the choice of reflections for refinement. *R*-factors based on *F*^2^ are statistically about twice as large as those based on *F*, and *R*- factors based on ALL data will be even larger.

### Fractional atomic coordinates and isotropic or equivalent isotropic displacement parameters (Å^2^)

**Table:**

	*x*	*y*	*z*	*U*_iso_*/*U*_eq_	
C1	0.4259 (5)	0.1591 (3)	0.1743 (3)	0.0497 (7)	
C2	0.3612 (5)	0.2317 (4)	0.2902 (3)	0.0606 (9)	
H2	0.2384	0.2966	0.2946	0.073*	
C3	0.4814 (6)	0.2060 (4)	0.3978 (3)	0.0729 (10)	
H3	0.4399	0.2533	0.4760	0.087*	
C4	0.6631 (6)	0.1104 (4)	0.3901 (3)	0.0688 (9)	
H4	0.7494	0.0940	0.4617	0.083*	
C5	0.7120 (5)	0.0399 (4)	0.2725 (3)	0.0616 (9)	
H5	0.8323	−0.0274	0.2667	0.074*	
C6	0.1290 (5)	0.2807 (4)	0.0538 (3)	0.0609 (9)	
H6A	0.1624	0.3874	0.1022	0.073*	
H6B	−0.0012	0.2245	0.0903	0.073*	
C7	0.0728 (5)	0.3080 (3)	−0.0806 (3)	0.0487 (7)	
C8	0.2253 (5)	0.3955 (4)	−0.1514 (3)	0.0616 (8)	
H8	0.3653	0.4367	−0.1155	0.074*	
C9	0.1723 (6)	0.4224 (4)	−0.2746 (3)	0.0702 (10)	
H9	0.2763	0.4823	−0.3210	0.084*	
C10	−0.0325 (6)	0.3617 (4)	−0.3297 (3)	0.0712 (10)	
H10	−0.0670	0.3785	−0.4135	0.085*	
C11	−0.1851 (6)	0.2764 (4)	−0.2600 (3)	0.0686 (10)	
H11	−0.3257	0.2362	−0.2958	0.082*	
C12	−0.1314 (5)	0.2501 (4)	−0.1371 (3)	0.0587 (8)	
H12	−0.2367	0.1911	−0.0910	0.070*	
N1	0.3211 (4)	0.1824 (3)	0.0631 (2)	0.0584 (7)	
H1A	0.3715	0.1364	−0.0055	0.070*	
N2	0.5982 (4)	0.0617 (3)	0.1662 (2)	0.0553 (7)	

### Atomic displacement parameters (Å^2^)

**Table:**

	*U*^11^	*U*^22^	*U*^33^	*U*^12^	*U*^13^	*U*^23^
C1	0.0601 (19)	0.0431 (16)	0.0460 (18)	0.0067 (15)	0.0030 (14)	0.0010 (13)
C2	0.075 (2)	0.0526 (19)	0.055 (2)	0.0143 (17)	0.0093 (16)	0.0009 (14)
C3	0.100 (3)	0.071 (2)	0.047 (2)	0.012 (2)	0.0090 (18)	−0.0041 (15)
C4	0.082 (2)	0.068 (2)	0.055 (2)	0.007 (2)	−0.0131 (17)	0.0029 (16)
C5	0.067 (2)	0.060 (2)	0.058 (2)	0.0129 (17)	−0.0012 (16)	0.0041 (15)
C6	0.063 (2)	0.060 (2)	0.061 (2)	0.0195 (17)	0.0017 (15)	0.0045 (15)
C7	0.0485 (18)	0.0403 (16)	0.0586 (19)	0.0120 (14)	0.0035 (14)	0.0031 (13)
C8	0.0514 (19)	0.059 (2)	0.073 (2)	0.0014 (16)	0.0016 (16)	0.0005 (16)
C9	0.077 (3)	0.063 (2)	0.071 (2)	−0.0001 (19)	0.0092 (19)	0.0130 (17)
C10	0.085 (3)	0.069 (2)	0.061 (2)	0.011 (2)	−0.0070 (19)	0.0054 (17)
C11	0.061 (2)	0.071 (2)	0.072 (2)	0.0066 (19)	−0.0091 (18)	−0.0034 (18)
C12	0.054 (2)	0.0490 (18)	0.071 (2)	−0.0012 (15)	0.0063 (16)	0.0000 (15)
N1	0.0655 (17)	0.0619 (17)	0.0499 (16)	0.0249 (14)	0.0036 (12)	−0.0015 (11)
N2	0.0594 (16)	0.0534 (15)	0.0547 (16)	0.0156 (13)	0.0023 (12)	0.0045 (11)

### Geometric parameters (Å, °)

**Table:**

C1—N2	1.338 (3)	C6—H6B	0.9700
C1—N1	1.354 (3)	C7—C12	1.368 (4)
C1—C2	1.391 (4)	C7—C8	1.381 (4)
C2—C3	1.369 (4)	C8—C9	1.376 (4)
C2—H2	0.9300	C8—H8	0.9300
C3—C4	1.374 (4)	C9—C10	1.370 (4)
C3—H3	0.9300	C9—H9	0.9300
C4—C5	1.373 (4)	C10—C11	1.365 (4)
C4—H4	0.9300	C10—H10	0.9300
C5—N2	1.331 (3)	C11—C12	1.372 (4)
C5—H5	0.9300	C11—H11	0.9300
C6—N1	1.444 (3)	C12—H12	0.9300
C6—C7	1.495 (4)	N1—H1A	0.8600
C6—H6A	0.9700		
			
N2—C1—N1	115.7 (2)	C12—C7—C6	121.6 (3)
N2—C1—C2	121.5 (3)	C8—C7—C6	120.7 (3)
N1—C1—C2	122.7 (3)	C9—C8—C7	120.7 (3)
C3—C2—C1	118.9 (3)	C9—C8—H8	119.7
C3—C2—H2	120.5	C7—C8—H8	119.7
C1—C2—H2	120.5	C10—C9—C8	120.6 (3)
C2—C3—C4	120.0 (3)	C10—C9—H9	119.7
C2—C3—H3	120.0	C8—C9—H9	119.7
C4—C3—H3	120.0	C11—C10—C9	119.1 (3)
C5—C4—C3	117.3 (3)	C11—C10—H10	120.4
C5—C4—H4	121.3	C9—C10—H10	120.4
C3—C4—H4	121.3	C10—C11—C12	120.0 (3)
N2—C5—C4	124.2 (3)	C10—C11—H11	120.0
N2—C5—H5	117.9	C12—C11—H11	120.0
C4—C5—H5	117.9	C7—C12—C11	121.9 (3)
N1—C6—C7	111.6 (2)	C7—C12—H12	119.1
N1—C6—H6A	109.3	C11—C12—H12	119.1
C7—C6—H6A	109.3	C1—N1—C6	123.4 (2)
N1—C6—H6B	109.3	C1—N1—H1A	118.3
C7—C6—H6B	109.3	C6—N1—H1A	118.3
H6A—C6—H6B	108.0	C5—N2—C1	118.0 (3)
C12—C7—C8	117.7 (3)		
			
N2—C1—C2—C3	1.8 (4)	C9—C10—C11—C12	−1.0 (5)
N1—C1—C2—C3	−177.8 (3)	C8—C7—C12—C11	0.2 (4)
C1—C2—C3—C4	0.3 (5)	C6—C7—C12—C11	179.2 (3)
C2—C3—C4—C5	−1.9 (5)	C10—C11—C12—C7	0.4 (5)
C3—C4—C5—N2	1.6 (5)	N2—C1—N1—C6	178.6 (2)
N1—C6—C7—C12	117.9 (3)	C2—C1—N1—C6	−1.7 (5)
N1—C6—C7—C8	−63.1 (4)	C7—C6—N1—C1	170.6 (3)
C12—C7—C8—C9	−0.2 (4)	C4—C5—N2—C1	0.3 (5)
C6—C7—C8—C9	−179.2 (3)	N1—C1—N2—C5	177.6 (3)
C7—C8—C9—C10	−0.5 (5)	C2—C1—N2—C5	−2.0 (4)
C8—C9—C10—C11	1.1 (5)		

### Hydrogen-bond geometry (Å, °)

**Table:**

*D*—H···*A*	*D*—H	H···*A*	*D*···*A*	*D*—H···*A*
N1—H1A···N2^i^	0.86	2.26	3.070 (3)	158

Symmetry codes: (i) −*x*+1, −*y*, −*z*.
